# Ontology-based specification and generation of search queries for post-market surveillance

**DOI:** 10.1186/s13326-019-0203-7

**Published:** 2019-05-30

**Authors:** Alexandr Uciteli, Stefan Kropf, Timo Weiland, Stefanie Meese, Klaus Graef, Sabrina Rohrer, Marc O. Schurr, Wolfram Bartussek, Christoph Goller, Philipp Blohm, Robin Seidel, Christian Bayer, Manuel Kernenbach, Kathrin Pfeiffer, Wolfgang Lauer, Jörg-Uwe Meyer, Michael Witte, Heinrich Herre

**Affiliations:** 1 Institute for Medical Informatics, Statistics and Epidemiology (IMISE), University of Leipzig, Leipzig, Germany; 2 novineon Healthcare Technology Partners GmbH, Tübingen, Germany; 3 OntoPort UG, Sulzbach, Germany; 4 IntraFind Software AG, München, Germany; 5 Federal Institute for Drugs and Medical Devices (BfArM), Bonn, Germany; 6 MT2IT GmbH & Co. KG, Ratzeburg, Germany

**Keywords:** Ontology, Information retrieval, Search queries, Spreadsheet-based ontology specification, Ontology generation, Post-market surveillance

## Abstract

**Background:**

The vigilant observation of medical devices during post-market surveillance (PMS) for identifying safety-relevant incidents is a non-trivial task. A wide range of sources has to be monitored in order to integrate all accessible data about the safety and performance of a medical device. PMS needs to be supported by an efficient search strategy and the possibility to create complex search queries by domain experts.

**Results:**

We use ontologies to support the specification of search queries and the preparation of the document corpus, which contains all relevant documents. In this paper, we present (1) the Search Ontology (SON) v2.0, (2) an Excel template for specifying search queries, and (3) the Search Ontology Generator (SONG), which generates complex queries out of the Excel template. Based on our approach, a service-oriented architecture was designed, which supports and assists domain experts during PMS. Comprehensive testing confirmed the correct execution of all SONG functions. The applicability of our method and of the developed tools was evaluated by domain experts. The test persons concordantly rated our solution after a short period of training as highly user-friendly, intuitive and well applicable for supporting PMS.

**Conclusions:**

The Search Ontology is a promising domain-independent approach to specify complex search queries. Our solution allows advanced searches for relevant documents in different domains using suitable domain ontologies.

## Background

According to the provisions of the current European Medical Device Directive 93/42/EEC [1, 2] and the new European Medical Device Regulation (applicable as from May 2020) [3], each manufacturer of medical devices has to set up a comprehensive system in order to identify, evaluate and integrate clinical data derived from the field application of a medical device after market access during post-market surveillance (PMS). Small and medium-sized enterprises in the field of medical devices are in need for operable systems for post-market data retrieval in order to enhance their PMS strategies and to be prepared for the growing requirements of the new European Medical Device Regulation.

A wide range of both internal (own quality management and compliant system) and external (scientific databases, medical congresses, internet-based knowledge & experiences, PMS by competent authorities) sources have to be monitored in order to integrate all accessible data about a medical device’s safety and performance. Currently, these detailed, continuous searches are still performed manually with a high input of time and personnel resources, making PMS a daunting task.

Literally, an employee has to type all search queries (e.g., “safety AND coronary stent”) into the input fields of a large number of different databases, congress sites and public search engines in order to gain a broad, unspecific hit list, interspersed with single, relevant content. This strategy of data retrieval reaches its limits assuming that several search strings have to be applied in order to monitor a whole range of medical devices, each featured by a variety of decisive search questions.

Additionally, each notable medical database uses its own, inherent syntax to specify search queries. This incompatibility between databases and the amount of different search tasks combined with the manual application to a multitude of databases results in low efficiency and a high potential for human error.

Setting up more complex queries in a simple manner by domain experts enables the definition of the topic of interest in a more specific way and circumvents the problem of retrieving irrelevant content.

In the OntoVigilance project (predecessor project of OntoPMS), we developed the Search Ontology (**SON**) v1.0 [4], a promising approach for an ontology-based specification of complex search queries. The modular architecture of the SON enables the re-use of ontology parts in different use cases as well as a quick and easy adaptation and extension of the ontology according to the specific requirements.

The developed Search Ontology and its application was evaluated in the OntoVigilance project by domain experts. This previous study has proven that the SON is a suitable method for modelling complex queries, but it needed to be optimized to face further requirements of domain experts. On the one hand, the structure of the Search Ontology can be simplified to improve the usability by domain experts. On the other hand, certain extensions are necessary to model all relevant query types. Based on these findings, we further developed and optimized the Search Ontology in the OntoPMS project. The Search Ontology v2.0 is presented in this paper. The SON is generic and can be used in any domain. For the application of the SON in a particular domain, it has to be extended by a domain ontology, such that the classes of the domain ontology are subclasses of the SON classes. We call such domain ontologies Domain-specific Search Ontologies (**dSON**). Whereas SON stands for exactly one ontology (namely the Search Ontology presented in this paper), dSON represents a class or a type of ontologies. Various Domain-specific Search Ontologies can be developed, such as dSON-PMS for the whole PMS domain or dSON-Niti for modelling queries regarding the material Nitinol. In addition, we developed an Excel template to specify the information required to create a dSON, which significantly simplifies the ontology development by domain experts. For the automatic generation of a dSON from the Excel template, we implemented the Search Ontology Generator (SONG). In contrast to the OntoQueryBuilder (OQB) [4], the SONG generates the complete dSON including all specified queries in the correct query syntax from the Excel template and provides it for external tools (e.g., the search engine). In this way, the search engine can get the complete dSON by accessing the SONG service without any requests or generating queries at search time.

## Methods

In Europe, market access of a medical device based on a CE-mark is granted after a successful so-called “conformity assessment process”, which includes passing an extensive series of tests, risks analyses and evaluations of clinical data on the medical device’s safety and efficacy. Nevertheless, the behaviour of a medical device over time in broad application can be investigated a priori only in a limited manner. Thus, PMS strategies are set up in order to retrieve and summarize application data of medical devices and to identify residual risks. Expressive search queries are needed to precisely define the topic of interest. The problem is that the manual creation of complex queries requires knowledge of the correct query syntax and is time-consuming and error-prone. This paper focusses on developing the Search Ontology Generator (SONG), a framework for ontology-based specification and generation of powerful search queries by domain experts with less effort and without knowing the query syntax.

### Example PMS question

Reports on unfavourable interactions between implant material and patient’s tissue have to be identified and evaluated in order to a) control residual and/or unexpected risk, b) determine vulnerable patient subpopulations and c) improve the respective medical implant or material, respectively.

An example PMS question could be to find out the unexpected side effects of the metal alloy Nitinol used for construction of endoscopic clipping systems. A search query has to be constructed covering the different aspects of the PMS question such as “unexpected complication”, “type of medical device” (endoscopic clipping system) and “used material” (Nitinol) by suitable search terms (e.g., “Nitinol”, “Nickel Titanium” and “NiTi” as synonyms of Nitinol or “unexpected complication”, “unforeseeable risk”, “adverse event”, etc. to describe the complication). Furthermore, it can be necessary to specify terms that should not appear in the text (negation), for instance, to exclude descriptions of preclinical tests or studies (e.g., terms like “animal”, “study” and “preclinical”). Finally, the desired terms have to be assembled to a valid search query using supported operators and brackets.

This example is used in the paper for further explanations.

### Approach

**Fig. 1:**
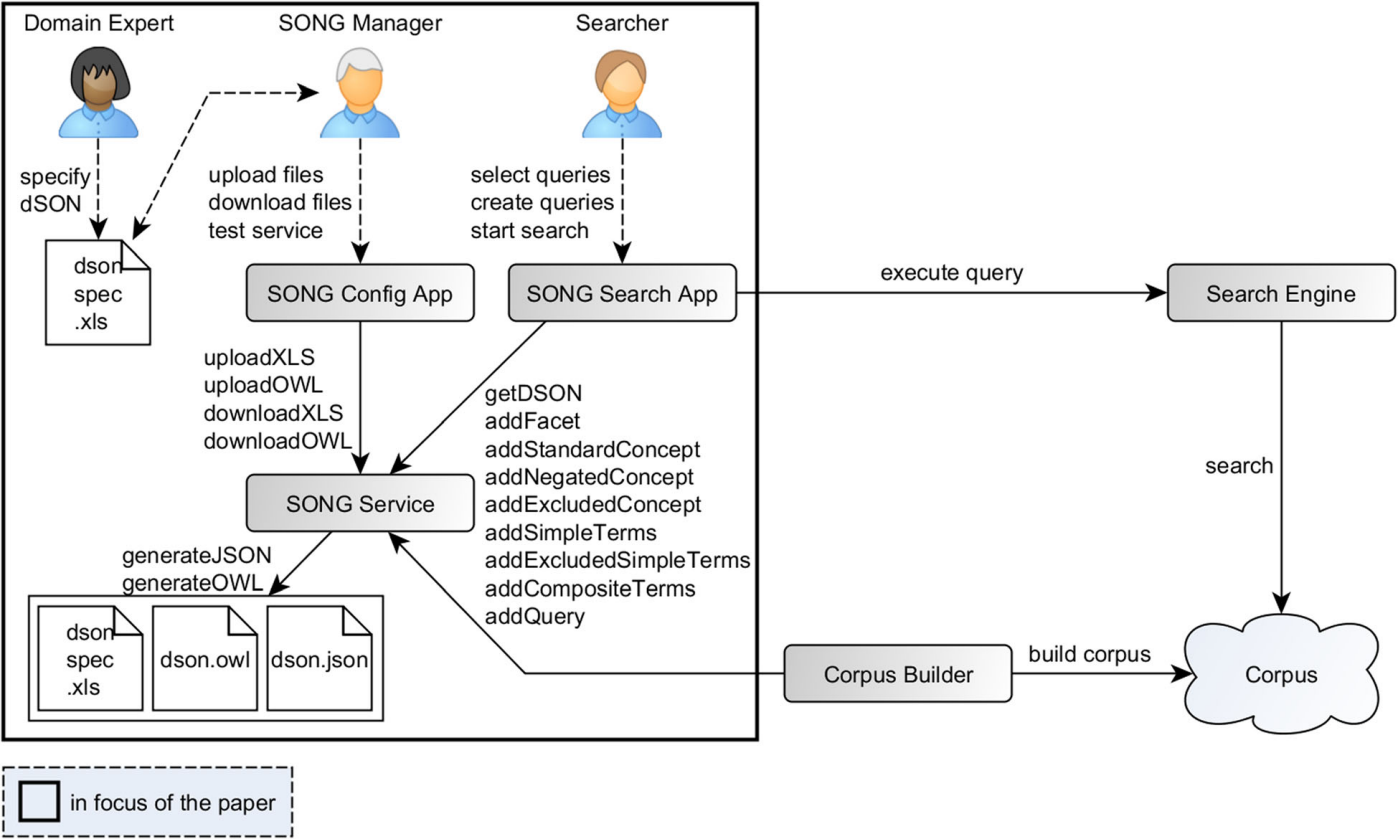
SONG and its application. The Domain Expert specifies the dSON in Excel. The SONG Manager uploads/downloads files and tests the service. The SONG Service generates the dSON (including complete search queries) and offers different methods for external applications. The Searcher uses the SONG Search App and can select desired concepts or queries for the execution by a search engine

Figure 1 illustrates the overall architecture of the SONG environment.

The Domain Expert specifies information required to generate a dSON (including search queries) using an easily applicable Excel template.

The SONG Manager uploads/downloads files and tests the service using the SONG Config App. The SONG service generates the dSON (in our example the dSON-Niti) in OWL and JSON format out of the Excel template, allows adding new entities to the ontology and provides the generated dSON for external tools, especially for search apps. After each file upload (Excel or OWL) or after an adding of a new entity, the new ontology (OWL and JSON) is generated.

The SONG manages the generation of OWL and JSON from Excel as well as JSON from OWL. The OWL format is used for a possible optimization of the generated ontology with an ontology editor or for an integration of external ontologies. Any ontology that contains concepts and their labels can be considered as a dSON and can be easily integrated with our approach. JSON is utilized for communication with external tools.

The Searcher uses the SONG Search App, which visualizes the dSON, and can select desired concepts or queries for the execution by a search engine.

The next sections introduce the two additional components, search engine and Corpus Builder that were used in the OntoPMS project in combination with SONG.

#### Search engine

The SONG can be used with any Lucene-based search engine for the generation of queries in the Lucene query syntax out of the Excel template. In addition, new expressive query operators were implemented in the OntoPMS project, which significantly extend the Lucene query syntax.

To identify risks or complications (for PMS) in unstructured documents, complex patterns have to be detected. Such patterns go beyond the standard capabilities of state-of-the-art search engines such as Elasticsearch [5]. Therefore, we extended these capabilities by creating our own search plugin, providing the required functionalities and improving search quality. The extension was realized as an Elasticsearch plugin and contains, among others, the following additional features: improved tokenization, lemmatization and word decomposition; build-in support for several normal forms / term types; improved quality for ambiguous searches; named entity, date and measurement recognition; additional search modes; NEAR operators.

In particular, the search modes and new search operators are extensively used in our dSONs to produce more precise queries. The search modes correspond to the different types of terms (exact [E], diacritics-normalized [D], lemmatized baseforms [B], compounds-parts [C]) that we use in our index. For example, with the query “MODE/E (SafeSet)” we only search for SafeSet with two upper case S. Words within a NEAR/n query must not have a token-distance greater than n. With NEAR/S and NEAR/P, words must occur within one sentence or one paragraph. These new NEAR-Operators can be combined and nested with other queries in an arbitrary way.

#### Corpus Builder

PMS requires information from several types of sources including proprietary manufacturer data and information from the web. Getting information from the web requires some knowledge on what to look for, how to look for it, how to access it and which parts to extract. Additionally, following the links found on a page identified by a given URL quickly turned out to be a potential trap, since the referenced pages may be completely out of scope. Therefore, we concluded that an ontology would help to define the scope and thereby control the automated data acquisition process.

For data acquisition, we developed the Corpus Builder [6, 7]. Its input component, the Prospector, metaphorically speaking, “roams” the internet in order to identify suitable data to “feed” the OntoPMS Corpus. To achieve this, it uses a set of special corpus queries, which are part of our dSON. Currently, the Prospector delivers its documents to a NLP pipeline, which analyses the contents to identify documents that are important to the respective projects and rejects (i.e. blacklist) documents that shall not be included into the OntoPMS corpus. This processing is done by a kind of control circuit. The “plant” of the feedback loop is controlled by a seed list, produced by the prospector and by the feedback component. It does the crawling and gathering of new URLs by following forward and backward links and reading the contents identified by the URLs. Then, the output is checked by the “sensor”. The sensor is controlled by the corpus queries and allows a deep analysis of the content. After that, questionable content is fed back to a splitter component which sorts out garbage (blacklist) and boosts domains with a high amount of documents we want to include (whitelist). URLs, based on those white listed domains are then included if not yet part of the seed list. If the output contains unwanted documents, we have to improve the corpus queries. Hence, we have a semi-supervised learning component, with which the manual part of supervision is made on the abstract level of ontologies. This enables us to change the behaviour of the corpus builder by changing the underlying ontology instead of changing the software.

## Results

### Search Ontology (SON) v2.0

The Search Ontology (SON) provides a general structure for all dSONs (the classes of the dSONs have to be subclasses of the SON classes; only relations and properties defined in the SON are allowed). This section presents the optimized Search Ontology v2.0, which contains some improvements compared to the v1.0. One of the advantages of the new version is that the SON-based dSONs can be specified in a specially developed Excel template rather than with an ontology editor. Furthermore, the keywords for the search (search terms) are directly associated with search concepts as annotations. In the v1.0, the search terms had to be defined as instances of the search term classes (with different labels) and linked to the search concepts using the property restrictions (based on the property *described_by*). Both aspects simplify the structure of the ontology. Other extensions include negated concepts and direct storage of queries in the ontology. The ontology contains 9 classes and 13 properties.

**Fig. 2:**
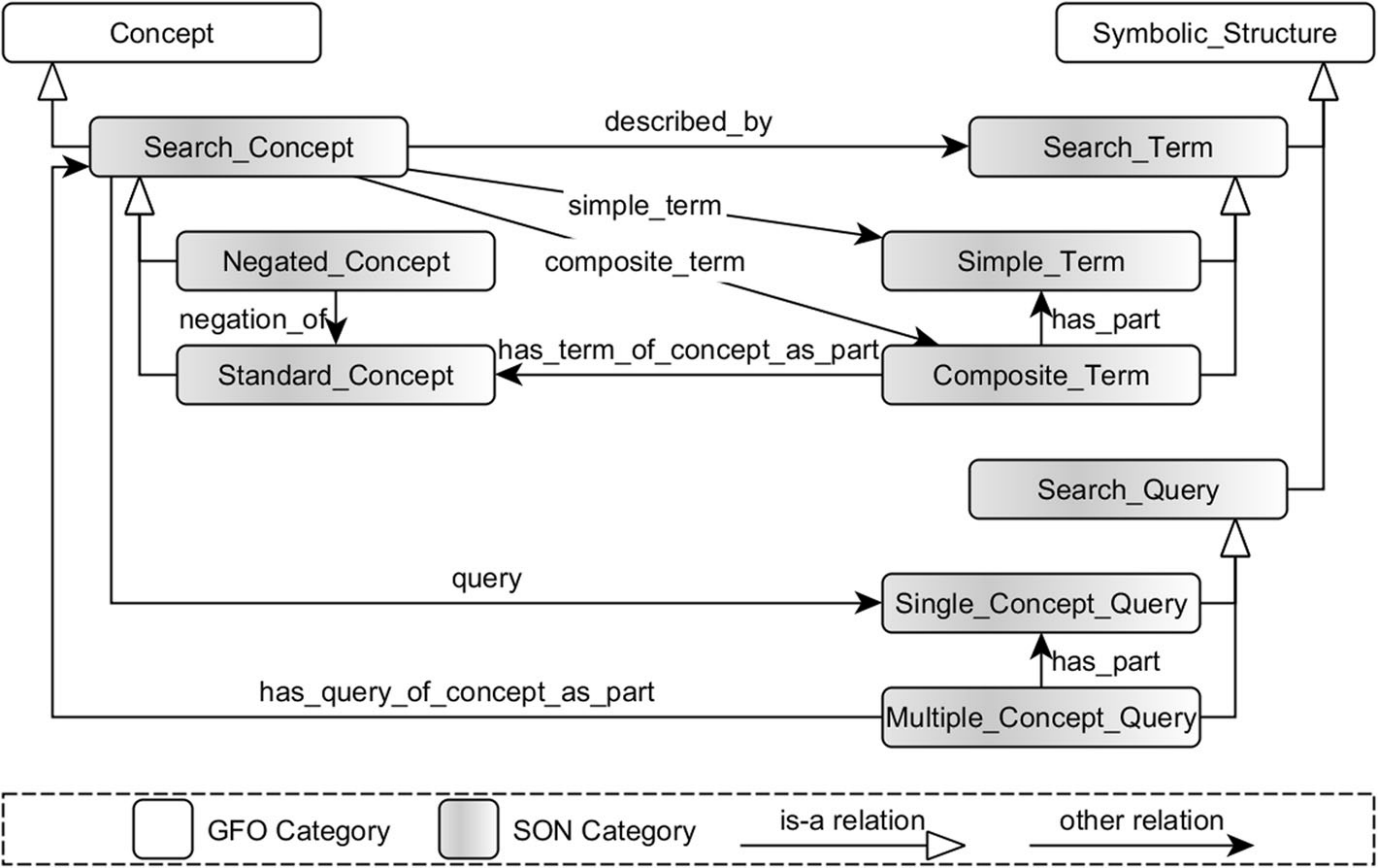
SON. The SON models three types of entities: search concepts, search terms, and search queries as well as several relations between them

The SON models three types of entities: search concepts, search terms, and search queries (Fig. 2). The search concepts are concepts (in the sense of General Formal Ontology, GFO [8, 9]), whose descriptions or designations have to be found in texts. The other two entities are symbolic structures (gfo: Symbolic_Structure) and serve to model single keywords or phrases of the concept description as well as queries.

#### Search terms

The search terms are descriptions or designations of search concepts. A distinction is made between simple and composite terms. The simple terms are either single words (e.g., “clip”, “Nitinol”) or fixed (defined by the user) phrases (e.g., “endoscopic clipping system”). The composite terms are combinations (*has_part*) of simple terms of two search concepts (*has_terms_of_concept_as_part*). They are defined by the user by choosing the two concepts (e.g., Unexpected and Complication) and are generated by the generator as an AND-connection of the OR-linked simple terms of the selected concepts (e.g., “(unexpected OR unforeseeable OR unknown) AND (complication OR failure OR incident)”).

#### Search concepts

The search concepts are described or designated by search terms (*described_by*, *simple_term*, *composite_term*). We distinguish between standard (e.g., Complication) and negated (e.g., No_Preclinical) concepts. While the terms of the standard concepts (e.g., “complication”, “failure”, “incident”) have to be contained in the resulting documents, the terms of the negated concepts (e.g., “animal”, “study”, “preclinical”) have to be excluded/negated. Each concept is additionally associated with a single concept query (see Search queries), which is used for the search for descriptions of the concept.

#### Search queries

The single concept query is an OR-connection of all terms (simple and composite) for a standard concept or a negated OR-connection of all terms for a negated concept. The query can additionally contain specified search operators and brackets. The multiple concept query is an AND-connection of single concept queries of selected concepts (*has_query_of_concept_as_part*).

### Excel template

**Fig. 3:**
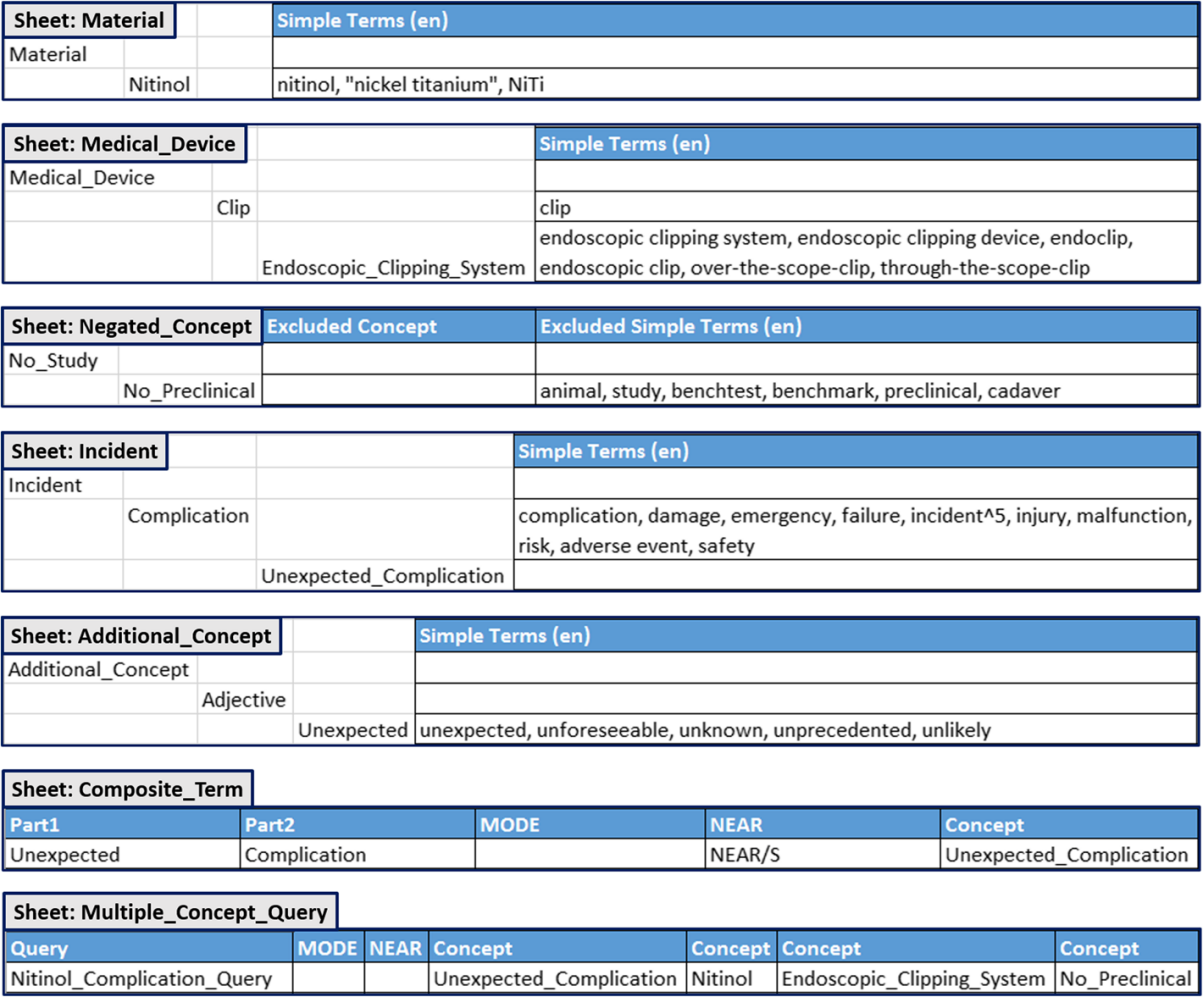
Excel template (excerpt). The different aspects of the PMS question such as “unexpected complication”, “type of medical device” and “used material” were modelled within the easily applicable Excel template

The design of the Excel template was derived from the SON structure. Sheets, tables and fields were created allowing the specification of all the information required for generating a valid dSON (including search queries). Figure 3 illustrates our example by screenshots of the several sheets.

The Excel template consists, on the one hand, of the predefined data sheets (Negated_Concept, Composite_Term and Multiple_Concept_Query) and, on the other hand, of the user-defined sheets (facet sheets) for the specification and classification of the search concepts and simple terms. For the observation of medical devices during PMS, we introduced among others the following facets: Medical_Device, Medical_Area, Medical_Problem, Incident, Material and Risk. In our example, the different facets of the search are represented by the Excel sheets Material, Medical_Device and Incident.

In every facet sheet, a subclass structure represents a categorization of the knowledge within the appropriate area. For instance, we subdivided medical devices in clip, stent, occluder and implant; moreover, these device categories can be subdivided into special types, e.g., endoscopic clipping system, PFO occluder, PDA occluder, and so on. Next to the nodes of the subclass hierarchy, the domain expert can enter simple terms; two columns (“Simple Terms (en)” and “Simple Terms (de)”) are used for enabling the separation of English and German simple terms. Several types of query operators, such as the wildcard or boost (e.g., incident^5), can be applied to simple terms in order to refine the search query.

For excluding documents that contain descriptions of certain concepts (e.g., complications in preclinical tests), the Negated_Concept sheet is used. The concept is specified (e.g., No_Preclinical) and described by simple terms to be excluded (e.g., “animal”, “study”, “preclinical”) in the column “Excluded Simple Terms”.

The Composite_Term sheet is used for the specification of terms based on other concepts. The composite terms for describing the search concept Unexpected_Complication (column: “Concept”) are combined from the simple terms of Unexpected (column: “part1”) and Complication (column: “part2”).

For the creation of complex (multiple concept) queries, which are based on the conjunction of queries of multiple search concepts (e.g., Unexpected_Complication, Nitinol, Endoscopic_Clipping_System and No_Preclinical), the Multiple_Concept_Query sheet is used. The name of the query is specified in the column “Query”. The definition of the improved MODE or NEAR (e.g., NEAR/S) operators (columns: “MODE” and “NEAR”) is possible for both, composite terms and multiple concept queries. Concepts that should be combined to a multiple concept query are specified in the columns “Concept”.

### Generating a dSON by SONG

By the generation of the ontology from the Excel template, the model presented in Fig. 2 is not applied one-to-one, but rather simplified. For example, simple terms and single concept queries are defined as annotations of the search concept classes.

Firstly, the SONG generates the class hierarchy. The search concept trees from the user-defined sheets (facet sheets) are placed under Search_Concept and the negated concept tree under Negated_Concept. Next, the simple terms (from the columns “Simple Terms (en)” and “Simple Terms (de)”) are linked to their concepts using the annotation property *simple_term*.

For each row in the Composite_Term table, a composite term class is generated as subclass of Composite_Term. The annotation properties *has_term_of_concept_as_part_1* and *has_term_of_concept_as_part_2* (shortly: *part_1* and *part_2*) are used to specify the two search concepts whose simple terms have to be put together. In addition, the specified MODE or NEAR operators are generated as annotations. Then, for each composite term class, the corresponding term is generated as an AND-connection of the OR-linked simple terms of the selected concepts, and is associated to the composite term class using the annotation property *query*. The possibly specified query operators for composite terms are taken into account in the correct syntax. The composite term classes are referenced in the search concept classes by the annotation property *composite_term*.

After that, the single concept queries of all search concepts are generated as an OR- connection for standard concepts or a negated OR-connection for negated concepts from all their terms, and are associated with the respective concept using the annotation property *query*. For negated concepts, the standard concepts can also be specified, whose terms have to be excluded (*excluded_concept*).

The multiple concept queries specified on the Multiple_Concept_Query sheet are generated as subclasses of Multiple_Concept_Query. The multiple concept query classes are associated with the concepts whose single concept queries are to be combined using the annotation property *has_query_of_concept_as_part* (shortly: *search_concept*). The single concept queries are AND-linked and stored using the annotation property *query*. Similar to composite terms, the possibly specified operators for queries are taken into account during the generation process.

**Fig. 4:**
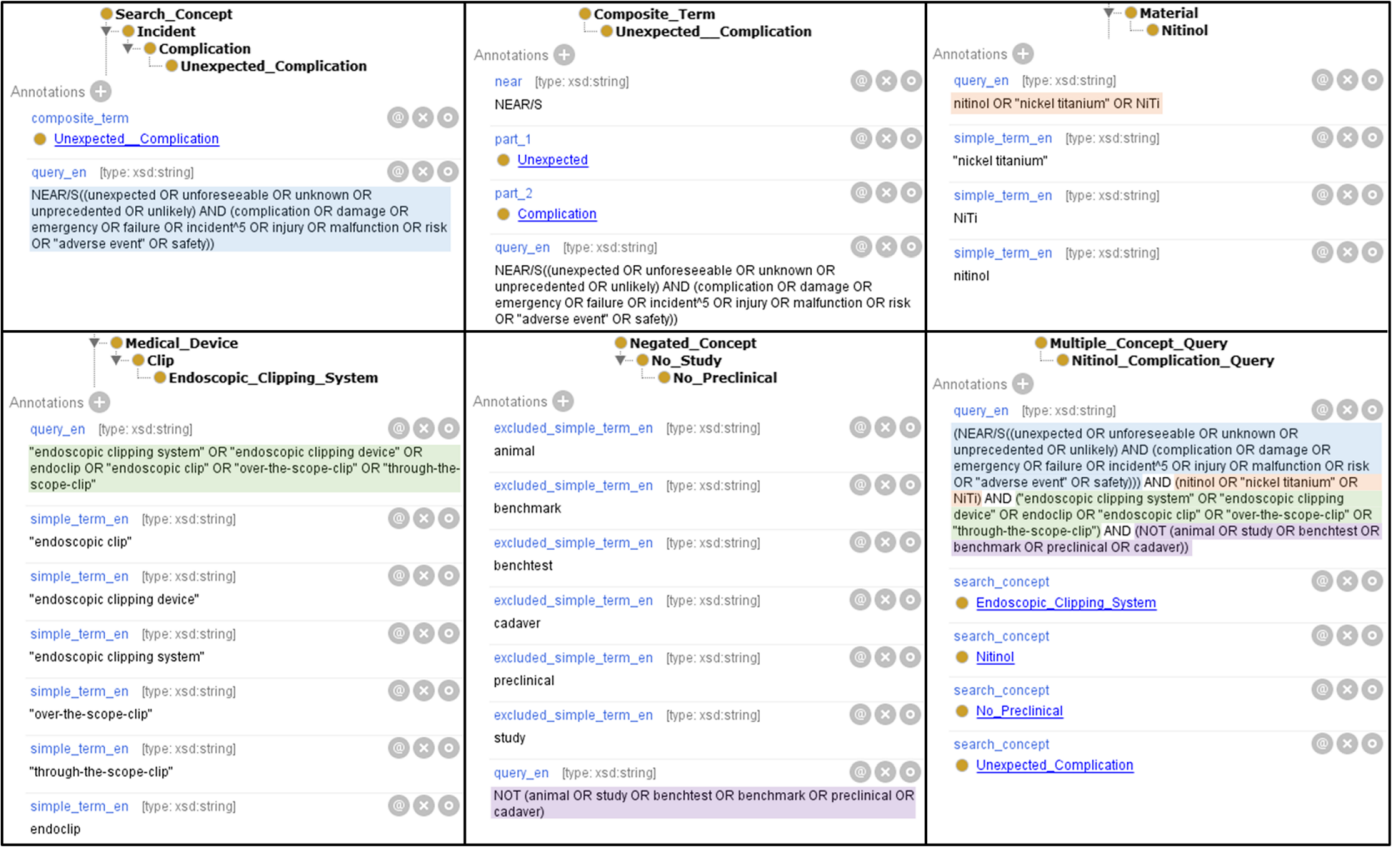
Parts of the dSON-Niti in Protégé. The upper part shows the search concept Unexpected_Complication, the composite term Unexpected__Complication and the search concept Nitinol. In the lower part, the search concept Endoscopic_Clipping_System, the negated concept No_Preclinical and the complete (multiple concept) query are illustrated

In Fig. 4, some parts of the generated dSON-Niti are illustrated. The upper part shows the search concept Unexpected_Complication, which is described by the composite term Unexpected__Complication (with two underscore characters), and the search concept Nitinol. In the lower left corner, the search concept Endoscopic_Clipping_System is presented. The lower middle part demonstrates the negated concept No_Preclinical, which is used for the exclusion of several simple terms. In the lower right corner, the complete (multiple concept) query is illustrated, which consists of different search concepts. In the annotation property *query* is the generated query, which can be executed by a search engine. The query parts (single concept queries) of the multiple concept query are highlighted.

### SONG Search App

**Fig. 5:**
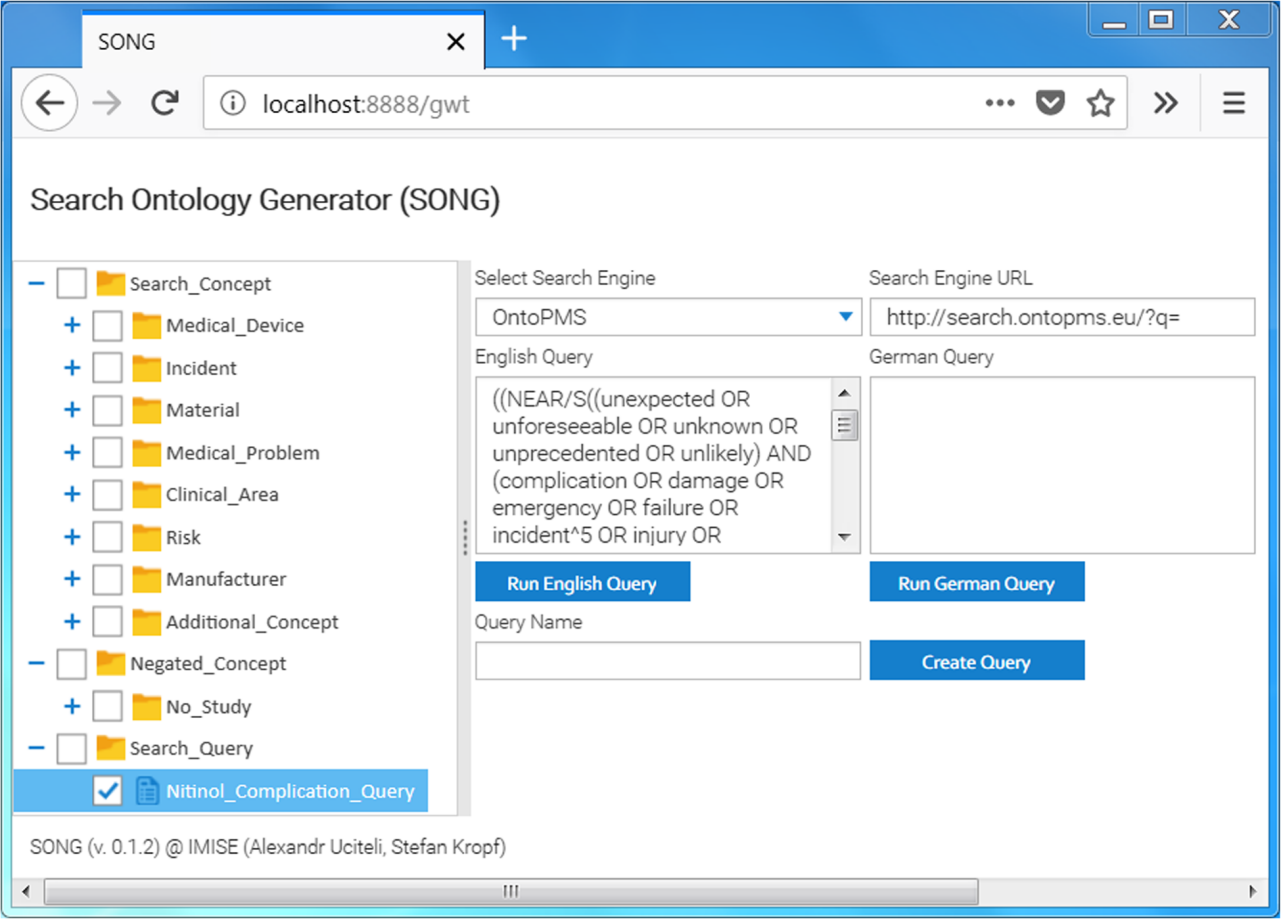
SONG Search App. The searcher clicks the checkboxes of desired concepts or multiple concept queries, selects a search engine and submits the generated query

**Fig. 6:**
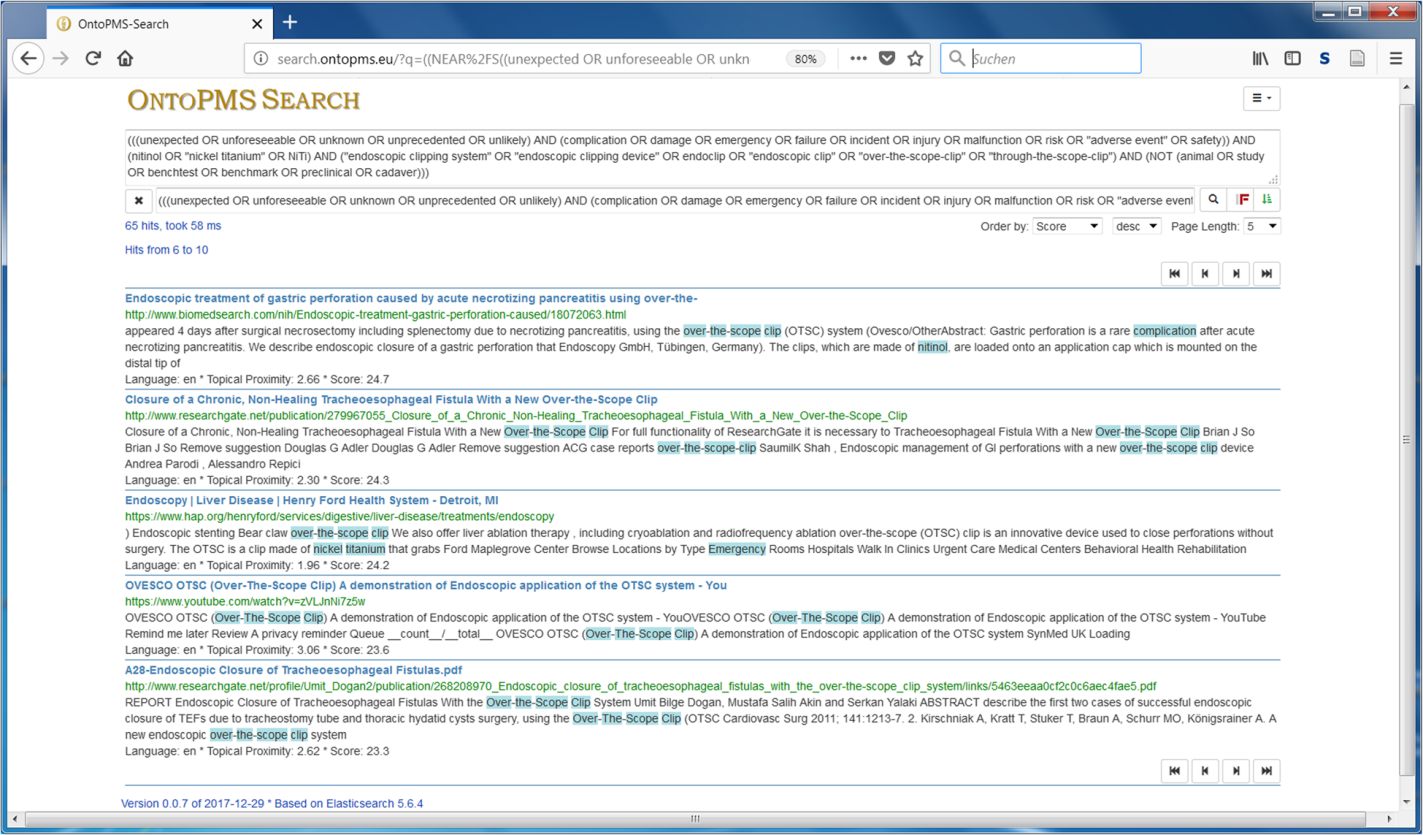
OntoPMS search results page. The search results of the OntoPMS search engine are displayed. Matching terms are highlighted. The query can be modified in the input field

**Fig. 7:**
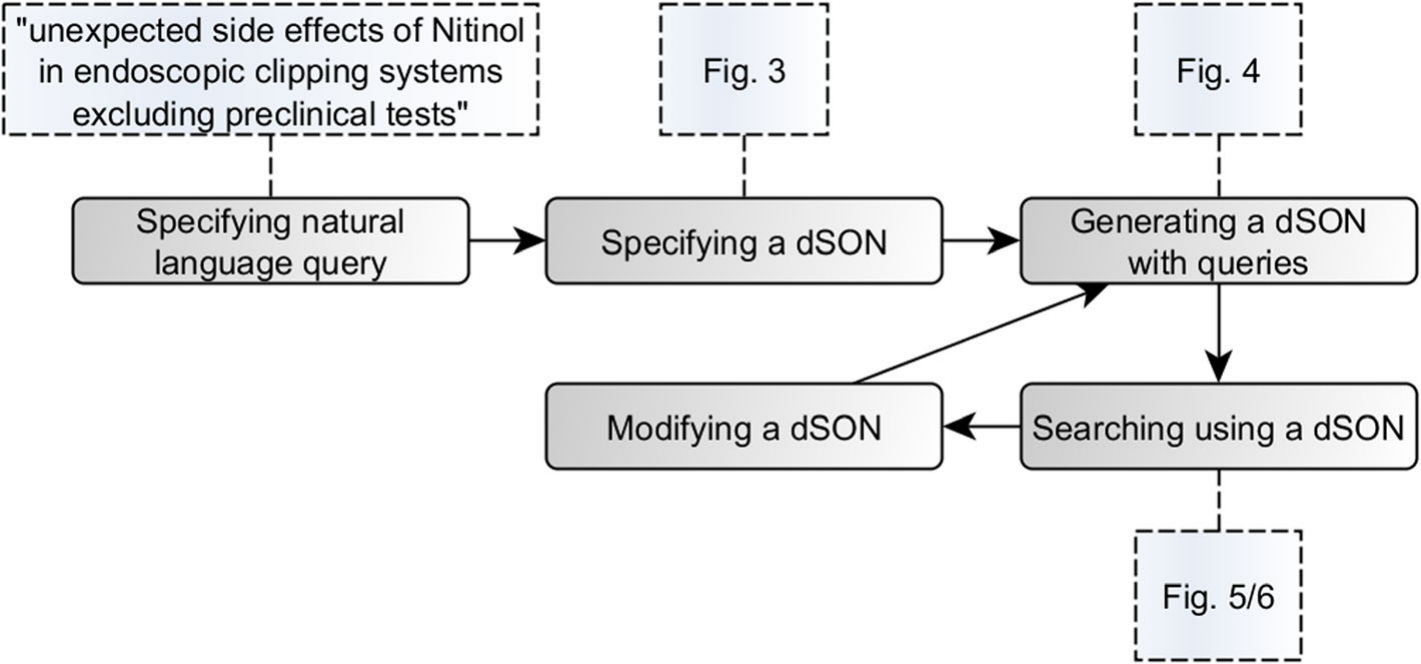
Ontology-based search pipeline. The figure summarizes the different steps of the ontology-based search. A recursive optimization of the query is possible

The SONG Search App accesses the SONG service and receives the generated dSON in JSON format (using the getDSON method of the service). The search concepts and multiple concept queries are visualized on the GUI of the app as a tree with checkable nodes (Fig. 5). The searcher can select desired concepts or multiple concept queries by checking the corresponding checkboxes. The overall query appears in the boxes “English Query” or “German Query” (depending on which language was used for specifying simple terms). The user can enter a search engine URL directly or can select an available search engine via the dropdown list. In Fig. 5, the OntoPMS search engine was selected. After pressing the buttons “Run English Query” or “Run German Query”, the query is forwarded to the search engine URL as GET parameter. On the website of the OntoPMS search engine (Fig. 6), the search results are displayed. Matching terms are highlighted. When minor changes are needed (e.g., deleting the NEAR or boost operators), the searcher can modify the query in the input field. However, the important changes of the query that should be available for further searches have to be made in Excel (see “Modifying dSON” in Fig. 7).

Additionally, the app supports the creation of new multiple concept queries. To achieve that, the user has to select desired concepts, to define the query name and to press the “Create Query” button. The app sends this information to the SONG service (using the method addQuery). After that, the SONG adds the new query to the ontology und transmits the updated dSON back to the GUI. The query is then available for further searches.

Figure 7 summarizes the different steps of the ontology-based search.

### Evaluation

Comprehensive testing confirmed the correct execution of all SONG functions. The applicability of our method and developed tools was evaluated by domain experts.

In the OntoVigilance project, development and editing of specific ontologies was conducted in Protégé [10]. For commercial use (in our case post-market surveillance purposes for medical devices), ontologies are rather dynamic constructs with a constant pace of re-editing to react upon changing search parameters. Designated key operators are Regulatory Affairs and/or Quality managers positioned at the respective departments of medical device manufacturers. Hence, the prototypical domain expert will be rather untrained in deep informatics/ontology processing. It turned out that Protégé’s basic functionality can be learned by a domain expert. However, the ontology editor was considered cumbersome and complex to operate. Consequently, in the OntoPMS project, an Editor easy to learn and at once applicable was formulated as an essential requirement.

Due to the broad application of MS office in business application, an Excel-based platform was highly appreciated by domain experts involved in the project. Three naïve domain experts were shortly instructed in the structure and function of the SON as well as the SON-based Excel template and equipped with a manual written by the SON developers.

Then each domain experts had to fulfill seven pre-defined tasks using the Excel template:

- Specification of a new facet “Equivalent product” (task 1.1)
- Specification of a new search concept “Clip XY” in the respective facet (task 1.2)
- Linking the search concept with pre-defined search terms in form of several simple terms (task 1.3)
- Specification of a new facet “Safety” (task 2.1)
- Specification of a new search concept “Unexpected Side Effect” in the respective facet (task 2.2)
- Linking the search concept with pre-defined search terms in form of composite terms (adjective-noun-phrase) (task 2.3)
- Specification of a negated concept “No Preclinical” with simple terms to be negated in the query (task 3)

Afterwards the test persons were introduced to the SONG and asked to generate a query to search for unexpected side effects of the Clip XY that are not related to preclinical tests (task 4).

Each test was accomplished successfully in a reasonable time. Table 1 provides the results of processing the tasks; Table 2 shows the time expenditure. Afterwards the test persons were briefly interrogated by preformed questions and asked to provide a general statement (Table 3). The test persons congruently rated the SON-based Excel template as clearly structured, featured by intuitive operator control logic and highly user-friendly. The Excel platform appears familiar. By application of the SONG, also complex queries can be generated quickly, transparent and reproducible. In comparison to a conventional periodic, manual data search, relevant content was identified by SONG-based data retrieval in a more convenient and comprehensive way.

**Table 1:** Successful task management

	task 1.1	task 1.2	task 1.3	task 2.1	task 2.2	task 2.3	task 3	task 4
Proband 1	2	2	2	2	2	2	2	2
Proband 2	2	2	2	2	2	2	1	2
Proband 3	2	2	2	2	2	1	2	2

0 = task not fulfilled

1 = task fulfilled; additional support required (Minor support was necessary in form of short explanations of the template structure while processing the tasks 2.3 and 3)

2 = task fulfilled without further support

**Table 2:** Expenditure of time in seconds

	task 1 complete	task 2 complete	task 3 complete	task 4 complete
Proband 1	72	60	13	20
Proband 2	47	60	20	19
Proband 3	61	89	16	22
Mean	60	70	16	20
SD	13	17	4	2

**Table 3:** Interview results

	Yes	No	Prefer not to say
Do you have any previous experiences with Ontology Editors?	0	3	0
Is Excel a common standard in regular work environment?	3	0	0
Is the structure of the ontology transparent in this set-up?	3	0	0
Do you rate Excel as feasible for ontology editing?	3	0	0
Is this set-up helpful for query generation?	3	0	0

In conclusion, the SONG framework significantly facilitates the creation of search queries. According to the domain experts in charge of the testing, the system is intuitive. Queries once generated can be saved and reused. This feature fosters transparency of a systematic search and repeatability, which is a legal imperative in the post-market surveillance of medical devices.

Additionally, BfArM’s Research group Safety of Medical Devices evaluated the applicability of SONG from a regulatory perspective giving a direct feedback from an assessor’s point of view. The evaluation was conducted based on the FDA coding system for medical device problems, which is implemented at BfArM to classify reported incidents. The evaluated tasks included not only adding, editing and deleting facets, concepts, simple and composite terms within an existing template but also generating complex queries, a task to be considered as highly important from a regulatory perspective. Since none of the assessors had been trained on the concept of ontologies before participating, the initial test results showed some minor but expected difficulties at first. Thereafter a fast learning process was observed, leading to desired performances within a very short period of time. Table 4 shows, that no help of any kind was needed (intuitional), whenever the complete information required for a given task was presented in one facet. Tasks including working with more than one facet or adding new information sometimes lead to mistakes, which could be corrected quickly and only by use of debug output (easy). Additional information was only required (explanation needed), when new concepts or composite terms had to be added. These kind of mistakes occurred only if it has been necessary to add new information while also consider consequences of this action to other facets. Notably no task needed to be explained more than once. Generating complex queries becomes a very easy task using this tool. One of the advantages of the current implementation is that it allows quick and easy modification of specified queries (e.g., by adding new simple terms) if the ontology needs to be altered due to a better understanding of the subject. The evaluation showed the fast increase in understanding the concept of search ontologies as well as the applicability of SONG to model the risk classification and to generate powerful search queries in a very systematic and efficient way.

**Table 4:** BfArM’s evaluation results

	Add	Edit	Delete
Facet	Easy	Intuitional	Intuitional
Simple terms	Easy	Intuitional	Intuitional
Concept	Explanation needed	Intuitional	Intuitional
Composite terms	Explanation needed	Easy	Intuitional
Multiple concept query	Intuitional	Intuitional	Intuitional

Intuitional = no help of any kind needed

Easy = quick correction of mistakes only by use of debug output

Explanation needed = additional information required (Explanation was needed only if it has been necessary to add new information while also consider consequences of this action to other facets. Notably no task needed to be explained more than once)

## Related work

### Ontology-based information retrieval

Since finding meaningful and intelligent information is difficult, there are different ontology information retrieval techniques and methods available [11]. In the wide world of semantic searches, the approach of this paper can be classified as Research Search [12], because we denoted search queries by concepts. Semantic searches are usually executed not on plain documents but on ontologies, which requires expensive manual annotation or natural language processing steps (NLP) for extracting semantic data out of the documents. After that step, the information of the documents is stored in a semantic knowledge base [13] or in a semantically enriched enhanced document index [14], on which semantic searches can be applied by using semantic retrieval languages such as SPARQL [15] or SeRQL [16]. The early TAMBIS project [17] provides a foresighted semantic search approach for accessing multiple bioinformatics databases, using a complex biological concept model for query formulation. Despite of semantic knowledge bases or structured data sources, the approach of this paper builds up on indexed documents which can be retrieved by complex Boolean expressions, which are difficult to construct [18]. Using ontologies as navigation tree structure in form of a Concept-based Information Retrieval Interface (CIRI) seems to be more effective than a direct interface (input field) [19].

GoPubMed [20] uses the Gene Ontology for search on PubMed. In contrast to our approach, the user is not able to increase the precision of the search by simply developing and using his own dSON, exactly tailored to his needs.

Textpresso [21] is a text-mining system for scientific literature. It implements categories of terms (an ontology) which can be used for a search on a database of articles. Regular expressions have to be created for each category to match the corresponding terms in the text and the documents have to be labelled according to the lexicon of the ontology. The documents are then indexed with respect to labels and words. Our solution does not use any in the ontology contained information for pre-processing or indexing of the documents. The ontology is constantly under development and is adapted by the domain experts to meet their current needs. Our approach does not require any additional pre-processing steps (e.g., labelling) as well as re-indexing the document collection when the ontology changes.

### Excel-based ontology development

Since ontology engineering is difficult for non-ontologists, there is a need for a rapid and collaborative ontology engineering methodology and easy to use tools [22]. The transformation of spreadsheets in OWL is already used in life science projects [23–25]; tools and plugins enable the population of OWL ontologies out of spreadsheet templates [26]. The template we developed differs in that way, that it is not intended for the ontology development in general based on modelling certain OWL constructs. Instead, our template is exactly tailored to the SON-based specification of dSONs in order to make its use by domain exerts as intuitive as possible.

## Discussion

The specification of complex search queries is a recurring task in different domains. The search for complications in the usage of medical devices within the post-market surveillance or the classification of the incident reports are only two examples in this area. Other examples are the patent examination or the search for relevant information in medical documents (e.g., discharge letter). Our solution is domain-independent and thus allows advanced search for relevant documents in different domains using suitable dSONs. The SON approach was also already successfully exploited for another use case in the medical domain to generate complex XPath expressions for querying archetype-based EHRs [27].

On the other hand, our approach is also generic regarding the used search engine. In the OntoPMS project, an Elasticsearch [5] based search engine was used, which was extended by developed plugins to provide novel expressive query operators. In addition, the document corpus was specially prepared and optimized for the PMS issue. However, other search engines can also be integrated relatively easily with our approach. The corresponding query syntax only needs to be implemented. For instance, scientists could use our solution to search in PubMed [28]. Nevertheless, not only scientists can benefit from our work. An office employee could model queries, which are relevant in his daily work and use them for the Google or Bing search.

Additionally, our solution supports the classification of documents (search results). When a query of a search concept delivers certain documents, they can be classified in this concept. The whole taxonomy of search concepts (part of the dSON) can then be considered as a classification of the entire result document set.

### Classification of search results

From the regulatory perspective of the German competent authority for risk assessment for medical devices, the Federal Institute for Drugs and Medical Devices (BfArM), there is an increasing demand to have the risk assessment process of critical incidents supported by intelligent IT solutions. With respect to the exponential increase in reported incidents with medical devices in Germany, specific dSONs for different aspects of the incident must be developed. These specific dSONs will allow the identification of similar incidents and thereby an automatic recommendation of classification of new incidents will become possible. The aspects currently in focus include dSONs for the resulting health problem, device problem, root cause and components, all of which are at present being developed on the basis of the FDA coding system, which is currently revised by the IMDRF working group on Adverse Event Terminology and Coding [29]. Using the SONG approach allows domain experts to easily create and modify the subsequent search concepts for each of the FDA/IMDRF Codes. Our approach will allow for accelerated risk classification, which in turn allows to create individual views of device-specific problems as well as to monitor the performance of different manufacturers within certain device groups such as hip implants, cardiac pacemakers, instruments for bone surgery or insulin pumps.

## Future work

Future work includes the application of our approach to other domains, especially the search for relevant information in medical documents as well as the integration of further search engines including the implementation of the corresponding query syntax. Since the PubMed search engine can process GET parameters, we can forward the generated query strings directly to PubMed. In future work, we planned to integrate fields such as “author”, “journal” and “title” as well as the MeSH terminology [30], including the subdivision in main and sub headings.

After the definition of a sufficiently extensive knowledge base in form of dSONs, ontology learning can be exploited for supporting a semi-automatic query creation.

## Conclusion

We presented the improved Search Ontology, a promising domain-independent approach to specify complex search queries. Our solution allows advanced search for relevant documents in different domains using suitable dSONs and supports an automatic classification of search results. The second version of the Search Ontology includes enhancements such as the inclusion of new search operators, negated concepts and direct storage of generated queries in the ontology. For easier handling by non-ontologists, we developed an Excel template, which facilitates a SON-based specification of dSONs without the usage of ontology editors or knowing the query syntax. A service-oriented architecture was introduced; in the core of the architecture stands the Search Ontology Generator (SONG), which provides methods for an access by search engines as well as dSON administration methods. By the enhancement of the SON, the Excel template, the SONG Search App and the service-oriented architecture, we improved the access to the Search Ontology for domain experts and external tools.

## Data Availability

The datasets generated and/or analyzed during the current study as well as the tools developed and further material used in the OntoPMS project are not publicly available. They contain information that could compromise research participants consent and contrast with the projects funding objective with the aim of strengthening the innovation capacity of small and medium sized enterprises in Germany. However, are available from the corresponding author on reasonable request.

## Abbreviations

dSON: Domain-specific Search Ontology
PMS: Post-market surveillance
SON: Search Ontology
SONG: Search Ontology Generator
